# Validation of a new t2* algorithm and its uncertainty value for cardiac and liver iron load determination from MRI magnitude images

**DOI:** 10.1002/mrm.25767

**Published:** 2015-05-22

**Authors:** Sebastian Bidhult, Christos G. Xanthis, Love Lindau Liljekvist, Gerald Greil, Eike Nagel, Anthony H. Aletras, Einar Heiberg, Erik Hedström

**Affiliations:** ^1^ Lund Cardiac MR GroupDepartment of Clinical PhysiologyClinical Sciences Lund, Lund University, Skåne University HospitalLundSweden; ^2^ Department of Biomedical EngineeringFaculty of EngineeringLund UniversitySweden; ^3^ Department of Computer Science and Biomedical InformaticsUniversity of ThessalyLamiaGreece; ^4^ Division of Imaging Sciences and Biomedical EngineeringKing's College LondonLondonUnited Kingdom; ^5^ BHF Centre of Research Excellence and NIHR Biomedical Research Centre at Guy's and St Thomas' NHS Foundation Trusts and King's College LondonLondonUnited Kingdom; ^6^ Laboratory of Medical InformaticsSchool of MedicineAristotle University of ThessalonikiGreece; ^7^ Department of Diagnostic RadiologyClinical Sciences Lund, Lund University, Skåne University HospitalLundSweden

**Keywords:** MRI relaxometry, iron‐load, offline image processing, validation, uncertainty estimation

## Abstract

**Purpose:**

To validate an automatic algorithm for offline T2* measurements, providing robust, vendor‐independent T2*, and uncertainty estimates for iron load quantification in the heart and liver using clinically available imaging sequences.

**Methods:**

A T2* region of interest (ROI)‐based algorithm was developed for robustness in an offline setting. Phantom imaging was performed on a 1.5 Tesla system, with clinically available multiecho gradient‐recalled‐echo (GRE) sequences for cardiac and liver imaging. A T2* single‐echo GRE sequence was used as reference. Simulations were performed to assess accuracy and precision from 2000 measurements. Inter‐ and intraobserver variability was obtained in a patient study (n = 23).

**Results:**

Simulations: Accuracy, in terms of the mean differences between the proposed method and true T2* ranged from 0–0.73 ms. Precision, in terms of confidence intervals of repeated measurements, was 0.06–4.74 ms showing agreement between the proposed uncertainty estimate and simulations. Phantom study: Bias and variability were 0.26 ± 4.23 ms (cardiac sequence) and −0.23 ± 1.69 ms (liver sequence). Patient study: Intraobserver variability was similar for experienced and inexperienced observers (0.03 ± 1.44 ms versus 0.16 ± 2.33 ms). Interobserver variability was 1.0 ± 3.77 ms for the heart and −0.52 ± 2.75 ms for the liver.

**Conclusion:**

The proposed algorithm was shown to provide robust T2* measurements and uncertainty estimates over the range of clinically relevant T2* values. Magn Reson Med, 2015. © 2015 The Authors. Magnetic Resonance in Medicine published by Wiley Periodicals, Inc. on behalf of International Society for Magnetic Resonance in Medicine. This is an open access article under the terms of the Creative Commons Attribution License, which permits use, distribution and reproduction in any medium, provided the original work is properly cited. **Magn Reson Med 75:1717–1729, 2016. © 2015 The Authors. Magnetic Resonance in Medicine published by Wiley Periodicals, Inc. on behalf of International Society for Magnetic Resonance.**

## INTRODUCTION

Organ failure caused by iron overload is a major cause of death in patients with iron load disease. Accurate quantification of organ iron load has been shown useful in tailoring the therapy for such patients 1. MR imaging is used as the current reference standard to assess iron load in different organs. It is noninvasive, has documented high reliability and has been validated to biopsies in the heart and liver 2, 3, 4, 5, 6, 7.

Different methods for quantification of iron load by MR T2* are generally used 7, 8, 9. T2* measurements can be performed from signal averages in a delineated region of interest (ROI) or on a pixel by pixel basis. Differences between methods are related to what is included in the ROI, the model used for curve fitting and applied echo times (TEs). In the presence of zero‐mean Gaussian noise, a standard least‐squares (LSQ) estimator is considered optimal 10. However, the non‐Gaussian noise found in magnitude MR images 11, 12 introduces a bias in the T2* measurement which depends on the signal‐to‐noise ratio (SNR). To reduce impact of noise on T2*, an exponential fit combined with a constant offset 13 and automatic truncation of data points 14 have been proposed. In addition, noise‐correction schemes for single‐channel coils 15 and root‐sum‐square (RSS) reconstruction of phased‐array coils 16 were recently introduced to further reduce noise‐bias.

Validated inline methods may simplify iron load measurements and improves clinical availability. However, robust offline T2* methods may have an important role in multivendor settings. The maximum likelihood estimate (MLE) method 17, 18, 19 is currently available for inline processing in some vendors and if the noise statistics are known, it is the optimal estimation method in terms of variance 17. However, noise statistics of an image‐set is rarely available offline without requiring additional user interactions such as manually defining background regions for noise estimation or specifying image reconstruction technique. Moreover, uncertainty estimates for the obtained T2* value are not provided by current ROI‐based methods.

Therefore, the purpose of this study was to introduce and validate an automatic algorithm for offline T2* measurements also providing uncertainty estimates for robust quantification of iron load in the heart and liver, optimized for a wide range of T2* values. The method was validated in numerical simulations, in a phantom study and was applied to cardiac and liver MR imaging in patients with known or suspected iron load disease.

## METHODS

Patients were included at two centers. The protocol and procedures comply with the Declaration of Helsinki, and were approved by the local research ethics committees. All studies were performed using 1.5 Tesla (T) Philips Achieva systems (Philips Healthcare, Best, The Netherlands). An overview of typical sequence parameters used in this study is provided in Table 1.

**Table 1:** Overview of Typical Sequence Parameters Used in This Study

Parameter	Clinical T2* (cardiac)	Clinical T2* (liver)	Phantom measurements			
			T2* sGRE (reference)	T2* mGRE (cardiac)	T2* mGRE (liver)	T1 MOLLI
Acquired voxel size [mm]	2 × 2	3 × 3	1.96 × 2.0	1.96 × 2.15	1.96 × 2.0	1.98 × 2.0
Slice thickness [mm]	10	10	8	10	10	10
Matrix size	160	116	112 × 106	112 × 102	112 × 110	116 × 90
FOV [mm]	320 × 320	348 × 348	220 × 212	220 × 220	220 × 220	230 × 180
TEs [ms]	2.5, 5.0, 7.5, 10.0, 12.5, 15.0, 17.5, 20.0, 22.5 and 25.0	1.2, 2.7, 4.2, 5.7, 7.2, 8.7, 10.2, 11.7, 13.2 and 14.7	1.34, 2, 3, 5, 7.5, 10, 12.5, 15, 20, 30, 40, 50, 75, 100, 150, 200, 300	2.5, 5, 7.5, 10, 12.5, 15, 17.5, 20, 22.5 and 25.0	1.3, 3.4, 5.5, 7.6, 9.7, 11.8, 13.9, 16, 18, 20.1	1.11
TR [ms]	26 ms	17 ms	6 × T1	26	38	2.4
Flip Angle	20°	20°	50°	20°	20°	35°
Parallel imaging	Factor 2 (SENSE)	No	No	Factor 2 (SENSE)	No	Factor 2 (SENSE)
Read‐out profile	Linear	Linear	Linear	Linear	Linear	Linear
Turbo factor	6	No turbo factor;	No turbo factor;	6	No turbo factor;	48
Preparation pulse:	DIR	SPIR	No preparation	DIR	SPIR	IR
Flow‐compensation	On	Off	Off	On	Off	On
T1 mapping scheme:	n/a	n/a	n/a	n/a	n/a	5(3s)3;

### Proposed T2* Analysis Method

We propose a new algorithm for T2* estimation in magnitude MR images called ADAPtive T2* estimation from combined Signal models (ADAPTS). It is a ROI‐based algorithm adapting the curve‐fitting approach to balance accuracy and precision. All image processing including the proposed algorithm was developed using MATLAB (v8.1.0.604, Mathworks) and was implemented in the medical image analysis software *Segment* 20, freely available for research purposes.

**Figure 1:**
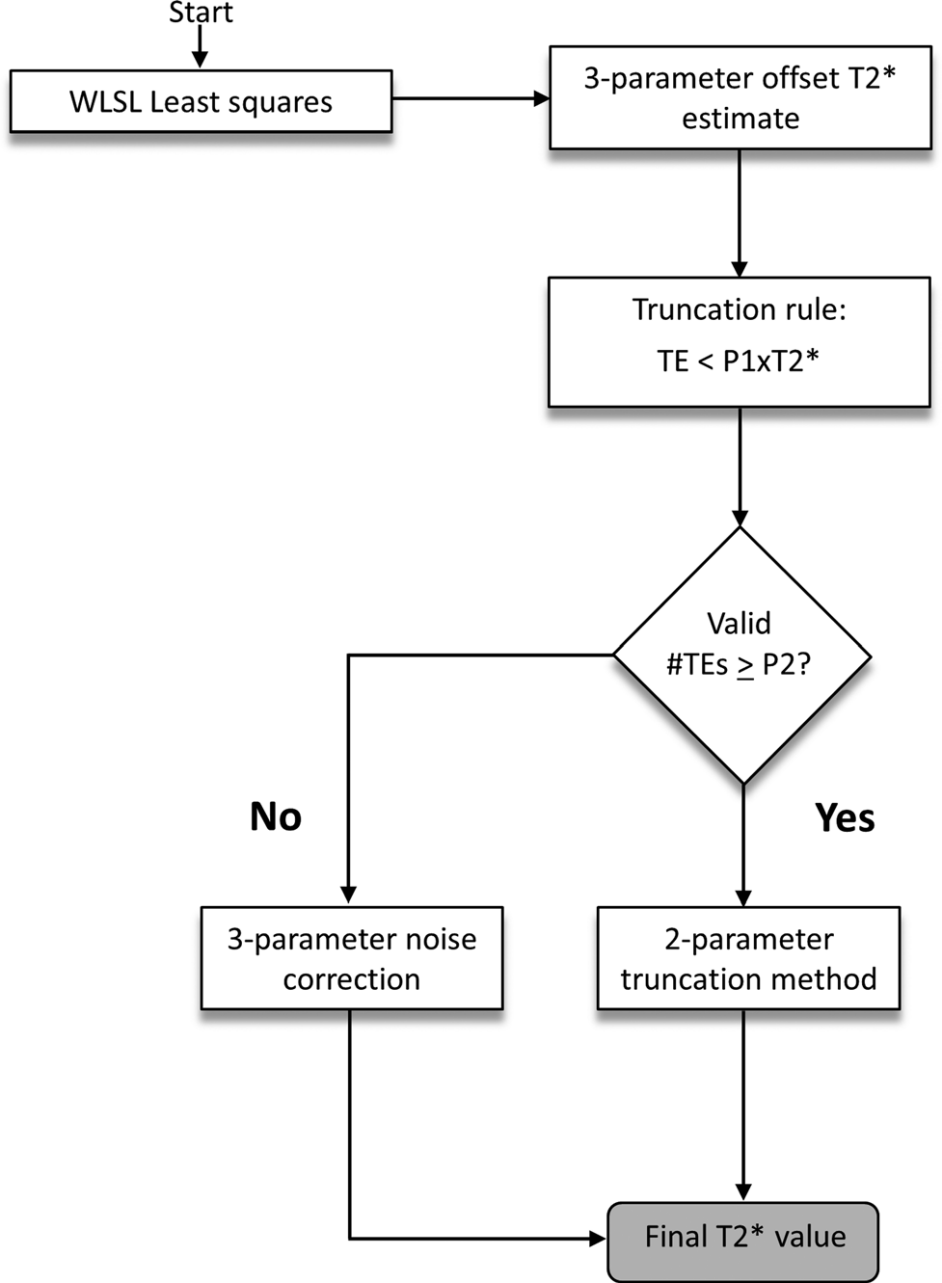
Overview of the ADAPTS algorithm.

An overview of the algorithm is provided in Figure 1. In all steps, the ROI average is used for curve‐fitting to increase SNR. The only manual interaction required is the delineation of a ROI. The first T2* estimate is a three‐parameter offset model 13, initialized by the weighted least‐squares on signal logarithm method (WLSL) 10. The signal model is shown in Eq. [1]:

(1) $$S\left(TE\right)=PD*e^{-TE/{T2}^{*}}+C.$$

The signal *S* depends on the TE, the proton density *PD* and an offset parameter *C* which approximates the noise‐floor. Compared with a two‐parameter monoexponential, shown in Eq. [2], The increased degree of freedom of a three‐parameter fit enables closer approximation of the measured signal 13.

(2) $$S\left(TE\right)=PD*e^{-TE/{T2}^{*}}.$$

This, however, makes three‐parameter models inherently sensitive to noise and over fitting to the obtained data points. The estimated offset parameter is used to approximate the noise plateau and instead of reporting the obtained T2* estimate (denoted $\hat{{T2}^{*}}$ in the remainder of this section) as the final T2* value, ADAPTS uses the initial fit for data‐truncation. TEs exceeding $P1*\hat{{T2}^{*}}$, where P1 is a nonzero constant, are excluded from the analysis and T2* is re‐estimated from a two‐parameter monoexponential fit (Eq. [2]) of remaining TE images, similar to the automatic truncation algorithm proposed by He et al 14. To refrain from extensive truncation which may lead to loss of precision, ADAPTS requires a minimum number of available TE images, a second constant P2, to proceed with the truncation method. If the number of valid TEs is below P2, ADAPTS assumes the number of remaining data points is insufficient for robust T2* estimation and switches to a noise‐correction approach, similar to the M2NCM method (Second‐Moment Noise‐Corrected Model), proposed by Feng et al 16. This method fits the observed signal in all available TE images to the expected value of the noncentral chi distribution in the presence of an underlying monoexponential decay:

(3) $$E\left[M^{2}\right]=S^{2}+2L\sigma^{2}.$$

Here, M denotes the measured signal contaminated with noise, S is the underlying exponential decay (Eq. [2]), σ is the noise standard deviation and  L the number of receiver coils in use. As previously proposed 16, the right term of Eq. [3] is estimated as a free parameter, resulting in a three‐parameter model. This removes the need for manual noise measurements. The motivation for balancing the amount of included parameters and data points in use by switching between signal models was to enable robust T2* estimation in a wide range of T2* values. The three‐parameter noise correction method is specifically designed to reduce noise bias in low SNR conditions and for T2* close to the minimum TE. However, the use of an additional free parameter may degrade precision for regions with high SNR where the noise bias is negligible. In these circumstances, a two‐parameter truncation method may result in improved precision. Although the signal models in ADAPTS have all been previously introduced, the proposed combination scheme is novel. All presented curve‐fitting methods used the Nelder Mead Simplex algorithm 21 for nonlinear optimization. Values for the constants *P1* and *P2* were optimized in the phantom study and in simulations, described in more detail below.

### Estimation of Uncertainty

To estimate uncertainty of the obtained T2* value, T2* was calculated in nonoverlapping, equally sized subregions. From the subregion ensemble of T2* values the 95% confidence interval (CI) size was estimated. The size of the subregions were defined as a fixed percentage of the ROI size to produce a near‐constant number of T2* values for each CI estimate. Due to the reduced number of pixels in the subregions compared with the ROI, the standard error of the mean (SEM) will increase for the pixel averages used for subregion T2* estimation. Assuming statistically independent pixels and a linear error propagation from the data‐points to the T2* estimate, a correction factor may account for the difference in standard error:

(4) $${SEM}_{subroi}=\frac{\sigma }{\sqrt{n}\;};\;{SEM}_{0}=\;\frac{\sigma }{\sqrt{N\;}}=\;\frac{\sqrt{n}}{\sqrt{N}}{SEM}_{subroi}$$

(5) $${\hat{\sigma }}_{T2^{*}}=\frac{\sqrt{n}}{\sqrt{N}}\;{\hat{\sigma }}_{T2^{*}subrois}.$$

Here, *N* denotes the number of independent pixels in the ROI and *n* denotes the number of independent pixels in a subregion. Although this measure does not directly correspond to the precision of the full‐ROI T2* estimate, it serves as an approximation which is further affected by T2* homogeneity and varying noise levels within the ROI. Compared with pixelwise T2* estimates, subregion T2* results in improved precision due to pixel averaging, which more closely resembles the original ROI estimate. ADAPTS reports the T2* 95% CI together with the coefficient of variation (CoV; defined as the standard deviation divided by the ROI T2* value). Based on the obtained CoV estimate the user may be advised to adjust the ROI delineation.

### Numerical Simulations

Numerical simulations were performed to assess accuracy and precision of ADAPTS and to evaluate reliability of the uncertainty estimate in relation to known T2* values. RSS reconstruction of 1, 6 and 32 receive‐coils was simulated with identical monoexponential T2* decay on the real and imaginary part of a complex signal. Zero‐mean Gaussian noise with predefined standard deviation (SD) σ was added to each channel and the magnitude signal was created by the root sum of squares operation:

(6) $$RSS=\sqrt{\sum_{l=1}^{L}M_{l}^{2}}$$

(7) $$M_{l}=\sqrt{{Im}_{l}^{2}+{Re}_{l}^{2}}$$

$M_{l}$ denotes the magnitude signal of receive‐channel l, *Im* denotes the imaginary signal component and *Re* the real component. The SNR was defined as *S_0_*/*σ*, where *S_0_* is the signal intensity at TE = 0. The mean of 40 independent signals was averaged before curve‐fitting to simulate ROI‐averaging. A T2* range of 1–50 ms for SNR = 15 was simulated. TEs corresponding to the clinical T2* cardiac and liver sequences were used with TE ranges of 2.5–25 ms and 1–20 ms, respectively. T2* values below the minimum TE were not simulated for either sequence. This resulted in simulated T2* ranges for cardiac and liver of 2.2–50 ms and 1–50 ms, respectively. Simulations were repeated 2000 times to evaluate accuracy and precision of ADAPTS. From the 2000 repetitions, the mean and 95% CI of the ADAPTS T2* calculation were computed for each simulated T2* value.

In addition to ADAPTS, a two‐parameter version of the noise correction method M2NCM was simulated which takes the true noise standard deviation as input. This enabled a comparison with a near‐optimal method. The optimized parameters P1 and P2, previously derived from phantom measurements, were refined in simulations and accuracy and precision of ADAPTS was compared with each of the two included signal models (two‐parameter automatic truncation and three‐parameter noise‐correction methods).

Reliability of ADAPTS uncertainty estimate was evaluated by comparing a total of 2000 CI estimates to the CI of the 2000 ADAPTS T2* ROI estimates. This procedure was repeated for multiple subregion sizes and ROI‐sizes to optimize the CI estimates. Simulated ROI‐sizes and region‐sizes was (40, 100, 200, 400) pixels and (4%, 6%, 8%, 10%, 12%, 15%, 20% and 25%, respectively). Simulated ROI‐size for evaluating the optimal subregion size was 40 pixels.

### Phantom Study

Twelve 300 mL gel‐phantoms with T2*/T1 values corresponding to iron overloaded myocardium 22 were used for validation. The phantoms consisted of a mixture of water, agarose, gadolinium (DOTAREM; Guerbet, France) and a SPIO Ferumoxsil contrast agent solution (LUMIREM; Guerbet, France). The concentrations of gadolinium and SPIO contrast‐agent were varied to alter T1 and T2* while the agarose concentration was kept constant. Each phantom was scanned separately and was submerged in a water‐filled container before imaging to reduce potential susceptibility artifacts.

#### Phantom Imaging

Phantom imaging was performed using a six‐channel SENSE head‐coil. Accuracy and precision of ADAPTS were evaluated in two clinically used, single breath‐hold, multiecho gradient‐recalled echo (mGRE) sequences for heart and liver imaging.

Accuracy of the clinical T2* sequences combined with ADAPTS was evaluated by comparison with a single‐echo gradient‐recalled echo (sGRE) reference sequence with (TR > 6 T_1_) to allow full longitudinal recovery between excitation pulses. T1 was measured in all phantoms with a Modified Look‐Locker Inversion‐Recovery (MOLLI) sequence using a 5(3s)3 scheme.

Precision was evaluated by calculating CIs in repeated measurements (n = 120 repetitions), in three phantoms for each sequence, and with target T2* selected to represent typical values seen in clinical imaging: (4 ms, 10 ms, 20 ms) and (2 ms, 8 ms, 15 ms) for the heart and liver sequences. All repetitions for a single phantom were performed in the same session, in direct sequence with a minimum pause of 7 s between repetitions. In addition, the repeated measurements were used to validate the proposed ADAPTS uncertainty estimate by direct comparison with the CIs obtained from the 120 repetitions, and the effect of varying the ADAPTS parameters P1 and P2 was evaluated in all combinations of a set of parameter values, specified in Figure 4. In total, 104 parameter configurations were evaluated and final values of parameters were chosen to maximize precision and minimize bias between ADAPTS and an inline MLE method. The sGRE method was not provided as reference in the parameter optimization to be able to use it for independent validation of accuracy for the chosen parameter set.

The sGRE reference sequence used a flip angle of 50° and typical TEs = (1.34 ms, 2 ms, 3 ms, 5 ms, 7.5 ms, 10 ms, 12.5 ms, 15 ms, 20 ms, 30 ms, 40 ms, 50 ms, 75 ms, 100 ms, 150 ms, 200 ms, 300 ms). The mGRE sequence for liver imaging used a flip angle of 20°, a repetition time of 38 ms, and TE = (1.3, 3.4, 5.5, 7.6, 9.7, 11.8, 13.9, 16, 18, 20.1 ms) and the mGRE sequence for cardiac imaging used a black blood DIR preparation scheme, a parallel imaging factor of 2 (SENSE), a flip angle of 20°, a repetition time of 26 ms and TEs = (2.5, 5, 7.5, 10, 12.5, 15, 17.5, 20, 22.5, and 25.0 ms). The MOLLI sequence used a repetition time TR/TE of 2.4/1.11 ms, and a flip angle of 35°. The shim volume was placed equivalently for all sequences in all phantoms and a simulated electrocardiogram was generated with a constant heart rate of 60 bpm. Further sequence details are found in Table 1.

#### Phantom Data Analysis

T1 in each phantom was measured from the provided inline T1 maps of MOLLI within a 3.1 cm^2^ ROI. T2* from the sGRE sequence was determined from the acquired magnitude images, by a two‐parameter monoexponential fit of the pixel‐mean using a near‐identical ROI compared to the one used for T1 measurements (ROI position was approximated due to subtle differences in resolution). The Nelder Mead Simplex algorithm (21) was used for nonlinear optimization and the initial starting values were obtained from the WLSL method 10. T2* from the two mGRE sequences were determined from the same 3.1 cm^2^ ROI using inline MLE maps 18, 23 and ADAPTS. The number of pixels within the ROIs for T2* estimation in phantoms was similar to the typical number of pixels in ROIs for heart and liver in the patient study.

### Patient Study

Twenty‐three patients (15 male; median age, 18 years; range, 1–69 years) with known or suspected iron load disease were included in this study. Written consent was given by the patients or, in case of minors, their guardians. MR images for determination of T2* values were collected as part of routine clinical iron load assessment.

#### Patient Imaging

For patient imaging, clinical mGRE sequences were used with a 5‐ or 32‐channel cardiac coil in two centers. Two similar sequences were used, one optimized for cardiac and one for liver imaging (Table 1). The two sequences differed in initial TE (2.5 ms versus 1.2 ms), and TE increment (2.5 ms versus 1.5 ms). Both sequences used the generally available SPIR fat suppression and minor parameter changes were allowed to adapt for patient heart rate and field of view (FOV). Fat suppression was applied to avoid impact of fat on T2* quantification, especially in the liver, since previous work has indicated improved precision using fat suppression in mGRE imaging 24.

To assess cardiac iron a mid‐ventricular slice was acquired using the clinical cardiac sequence. A black‐blood double‐inversion recovery (DIR) prepulse was used to decrease measurement error induced by blood contamination, and to enhance myocardial borders 25. Images were acquired within a single breath‐hold using parallel imaging factor 2 (SENSE) to improve image resolution. Acquisition was carried out at end‐diastole within a time window of approximately 110 ms per heartbeat. To assess liver iron, a midhepatic transversal slice was acquired using the clinical liver sequence.

Online reconstruction of T2* maps from an inline MLE method 18, 23 was automatically performed for comparison with inter‐ and intraobserver variability of ADAPTS. Furthermore, T2* determined from ADAPTS and MLE in patients were directly compared as a proxy to the reference standard sGRE sequence, based on the results from the phantom validation.

#### Patient Data Analysis

Data were anonymized and randomized for blinded analysis. The ROIs were manually drawn at two occasions, by two observers (14 years and no previous MR experience, respectively) for analysis of intra‐ and interobserver variability, also accounting for user experience.

The ROIs were drawn in the acquired images for evaluation of T2* in full thickness myocardial septum and in a homogenous area of the liver parenchyma, anteriorly if not prevented by vessels or susceptibility artifacts. These exact ROIs were copied to the inline‐constructed MLE T2* map to avoid measurement differences related to ROI delineation. The MLE image was reconstructed from the very same acquisition as the ADAPTS analysis. To assure adequate ROI placement for both ADAPTS and MLE the curve fit using ADAPTS was visually inspected and the ROI redrawn in case of obvious incorrect placement. Software advice based on CoV for re‐evaluation of delineation was also considered. Motion correction was not performed in the current study, as motion between images acquired within the same breath‐hold was not detected.

### Statistics

Statistical analyses were performed in MATLAB (v8.1.0.604, Mathworks). By default, statistical measures for the patient study were obtained from the experienced observer. Bias and variability are presented as mean ± 1.96 SD and median (range) was used where appropriate. Bland‐Altman analysis 26 was used to compare methods and to analyze intraobserver and inter‐observer variability in the patient study. Accuracy was defined as the obtained bias compared with a reference standard and the 95% CI was used to measure precision. In this study, CI is reported as the size of the 95% CI. A *P*‐value < 0.001 was used to define statistical significance.

## RESULTS

### Numerical Simulations

**Figure 2:**
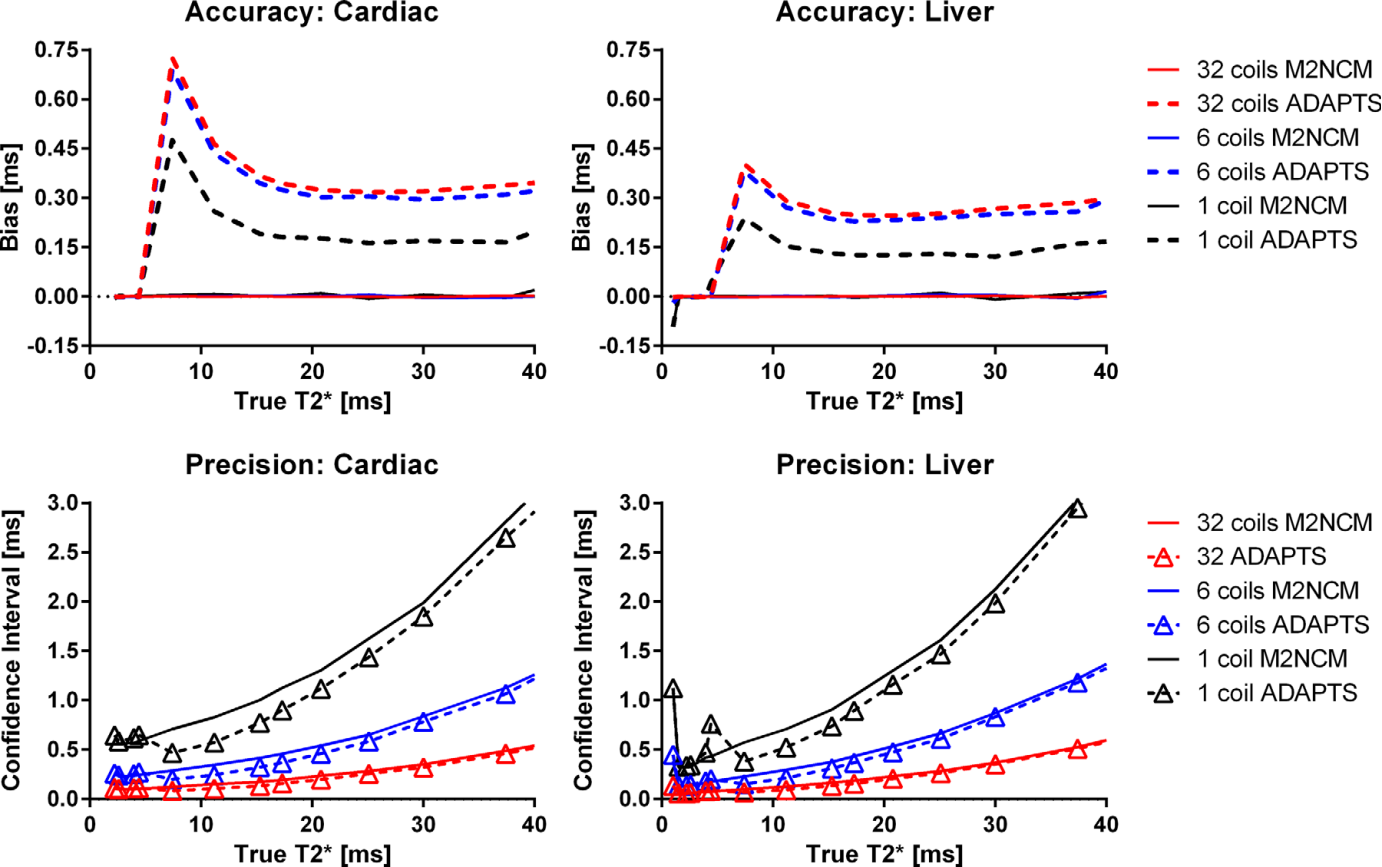
Accuracy and precision in numerical simulations. Solid lines indicate the M2NCM method and dashed lines show ADAPTS. Number of simulated coils are color‐coded. The upper panels show Accuracy in terms of mean differences between true T2* values, shown on the x‐axes. The lower panels show CIs from all simulation experiments (2000 repetitions), for each simulated T2* value. Within the clinically relevant range, the proposed method results in high accuracy and precision. The gradual decrease in precision with increasing T2* is most likely attributed to lack of available data‐points above 25 ms and 20 ms for cardiac and liver TEs, respectively, and is also seen in the near‐optimal noise‐corrected method. Simulated ROI size was 40 pixels.

**Figure 3:**
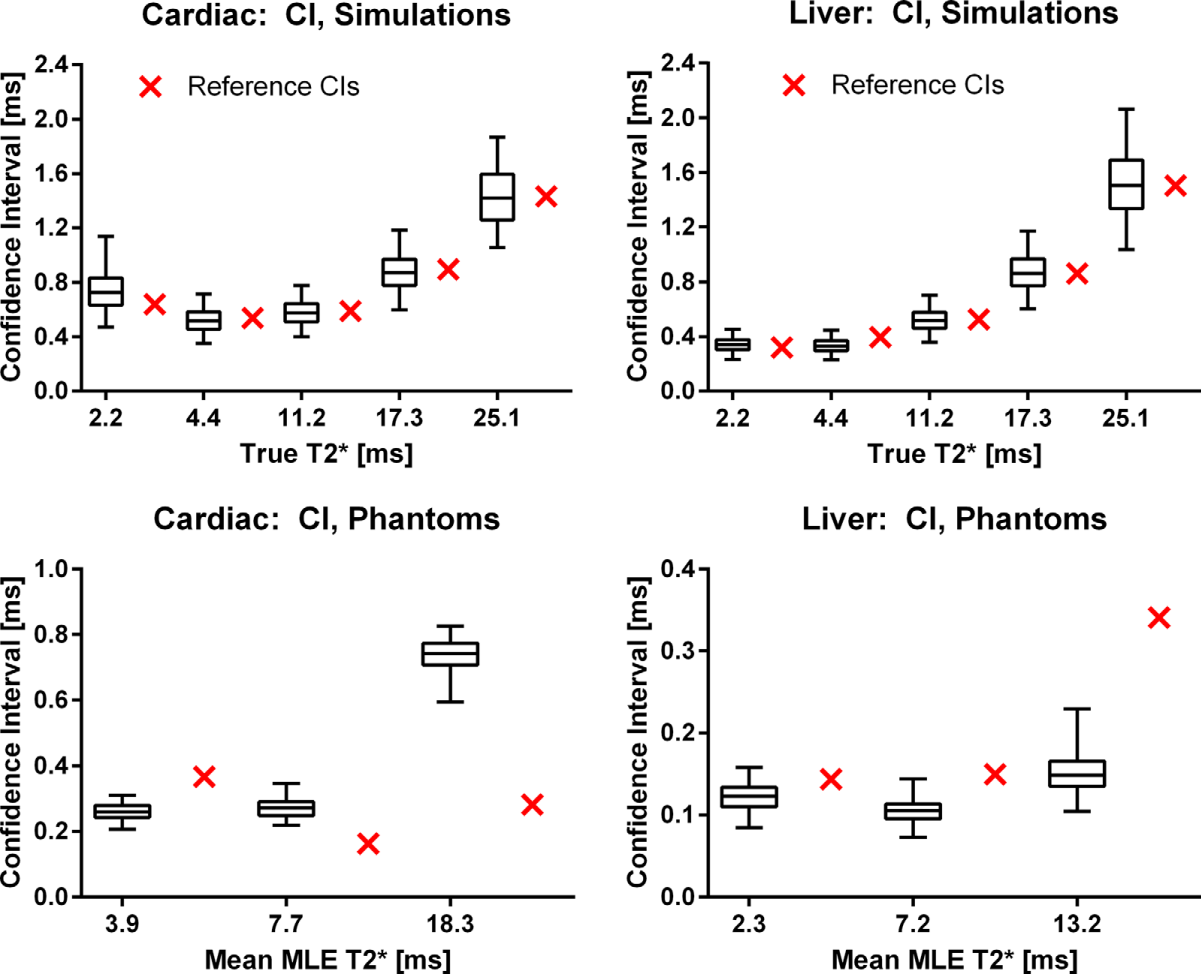
Box‐whiskers plots of the ADAPTS uncertainty estimate validation in simulations (top row) and repeated phantom measurements (bottom row). Simulations: 2.5% (bottom whiskers) and 97.5% (top whiskers) confidence limits from 2000 ADAPTS CI estimates in numerical simulations compared with the CIs obtained from the 2000 repetitions, shown as crosses directly to the right of each corresponding CI estimate. Boxes indicate first and third quartiles of the CI estimates and the horizontal line splitting the boxes shows the median. Note that the CI references (crosses outside boxes) all lie well within the confidence limits of the ADAPTS CI estimates for the simulated T2* values. Simulated ROI size was 40 pixels. Phantom study: 2.5% (bottom whiskers) and 97.5% (top whiskers) confidence limits from 120 ADAPTS CI estimations in repeated phantom scans, compared with the CIs calculated over all 120 repetitions (crosses directly to the right of the corresponding CI estimates). Limited overestimation and underestimation of CIs are observed.

**Figure 4:**
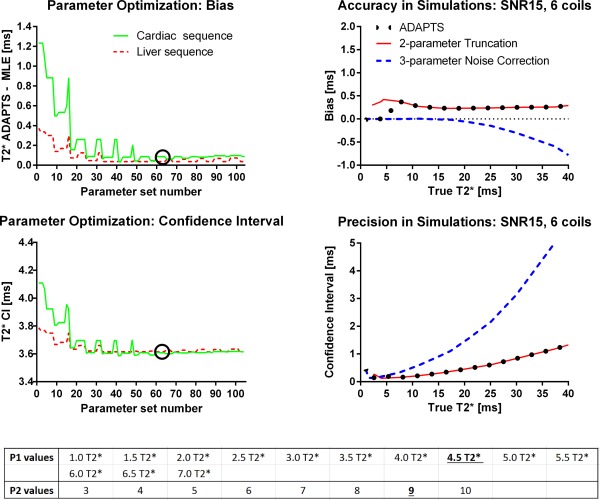
Parameter optimization from phantoms (left column and bottom table) and simulated optimal parameters of the ADAPTS method for the liver sequence TEs (right column). Parameter optimization: Top row shows how varying ADAPTS two parameters impacts bias, defined here as the mean difference between ADAPTS and the inline MLE method. Bottom row shows precision, measured as the CI size of 120 phantom measurements over different parameter values, together with the list of evaluated parameters. In both top and bottom rows, solid lines indicate measurements performed for the cardiac sequence and phantoms and the dashed lines indicate measurements from the liver sequence and phantoms. Circles show the selected parameter set used in the remaining parts of this study. The results indicate a robustness to variations in parameters above a threshold of approximately P1>3. Right column compares accuracy and precision of the optimized ADAPTS method with the two included signal models (truncation and noise‐correction) individually in simulations. The solid lines indicates the two‐parameter truncation method, the dashed line shows the three‐parameter noise correction method and the dotted line shows ADAPTS using the optimized parameter values. The optimized ADAPTS method balances low bias with maintained precision over the simulated T2* range. The shown simulations use TEs from the liver sequence, a SNR of 15, a ROI‐size of 40 and uses RSS reconstruction with six receive‐coils. The list of evaluated parameter values (bottom table) indicates the selected values of P1 and P2 with an underlined bold font.

Results from the simulation study are shown in Figures 2, 3, 4. Accuracy and precision for ADAPTS and the two‐parameter M2NCM method is found in Figure 2. The mean differences between ADAPTS and true T2* for TEs corresponding to the cardiac and liver clinical sequences ranged from 0 to 0.73 ms and 0 to 0.40 ms. In both cases, the largest mean difference was found in the 32‐coil simulation. Precision in terms of the 95% CI ranged from 0.08–4.30 ms for the cardiac TEs and 0.06–4.74 ms for the liver TEs. Mean differences for the two‐parameter M2NCM method ranged from 0 to 0.04 ms for the cardiac and 0 to 0.10 ms for the liver TEs. For M2NCM, the maximum mean difference was found for the single‐coil simulation. CIs were 0.05–1.97 ms and 0.02–2.27 ms for the cardiac and liver sequences. An increase in bias was observed for the ADAPTS method when the number of simulated coils increased. Precision was improved when the number of simulated coils were increased. Supporting Figure S1, which is available online, shows bias and CIs of the optimized parameters P1 and P2 in simulations. P2 = 9 was selected as optimal parameter value. A comparison of accuracy and precision for ADAPTS and the two implemented signal models for the liver sequence TEs is shown in Figure 4. The two‐parameter truncation method resulted in a limited but constant overestimation of T2* over the simulated range, while bias was minimal for the noise correction method for T2* below 20 ms. For T2* values above the maximum TE, an increasing underestimation was observed for the noise correction method.

The simulation results for ADAPTS uncertainty estimates are shown in Figure 3. Four percent was selected as the optimal subregion size, resulting in a mean bias and CI of 0.02 ms and 0.30 ms for all simulated ROI‐sizes. For all simulated T2* values, and TEs, the CI reference standard lies within the 2.5% and 97.5% confidence limits of the 2000 uncertainty estimates and the largest observed mean bias between the CI estimate and the CI reference was 0.10 ms. Supporting Figures S2 and S3 shows bias and CIs of the uncertainty estimate over varying ROI‐ and subregion‐sizes. A consistent improvement in precision (CI) was observed when decreasing the subregion size.

### Phantom Study

The T2* and T1 ranges of the 12 phantoms were 2.20–40.24 ms and 470–1012 ms according to the T2* reference standard (sGRE) and MOLLI T1. T1 values are in the range of myocardial tissue. T2* of the six phantoms used to evaluate precision was 2.3, 3.9, 7.2, 7.7, 13.2, and 18.3 ms, obtained from the inline MLE method. The results from the phantom validation of ADAPTS uncertainty estimates for both clinical sequences are shown in Figure 3 (bottom row). The maximum observed difference between ADAPTS uncertainty estimate and the reference CI (from 120 repetitions) was limited to 0.58 ms for the cardiac sequence, and 0.27 ms for the liver sequence.

**Figure 5:**
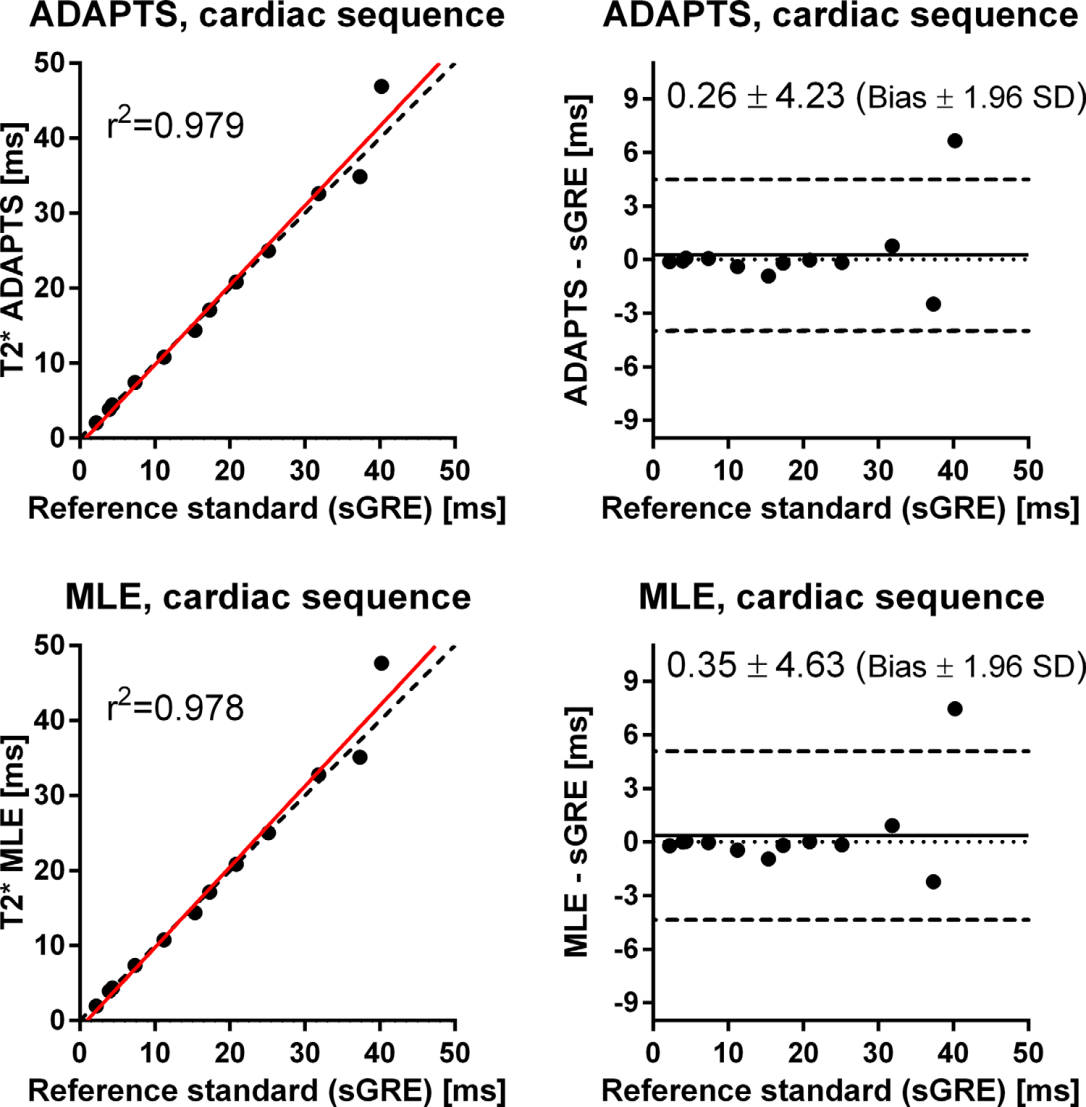
Scatter plots (left) and difference plots (right) of T2* by ADAPTS (top) and MLE (bottom) using the clinical cardiac sequence, compared with the T2* reference standard (sGRE) in phantoms. Scatter plots: solid lines indicate linear regression and dashed lines represent identity lines. Difference plots: Solid lines indicate bias and dashed lines represent bias ± 1.96 SD. T2* values by ADAPTS and MLE using the clinical cardiac mGRE sequence agree well with the reference standard sGRE over a wide range of T2* values.

**Figure 6:**
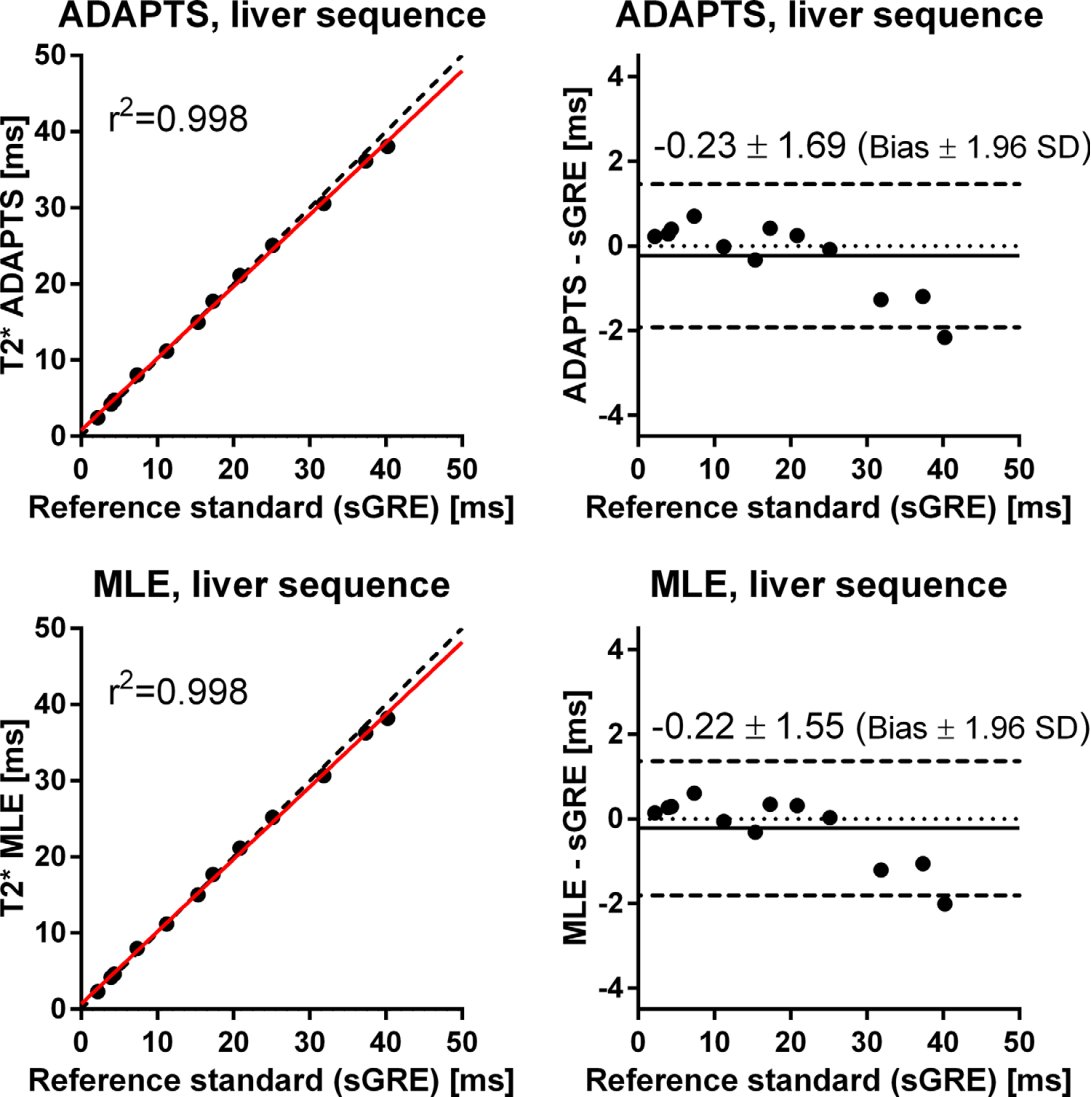
Scatter plots (left) and difference plots (right) of T2* by ADAPTS (top) and MLE (bottom) using the clinical liver sequence compared with the T2* reference standard sGRE in phantoms. Scatter plots: solid lines indicate linear regression and dashed lines represent identity lines. Difference plots: Solid lines indicate bias and dashed lines represent bias ± 1.96 SD. T2* values by ADAPTS and MLE using the clinical liver mGRE sequence agree well with the reference standard sGRE over a wide range of T2* values.

The results from the parameter optimization of ADAPTS are shown in Figure 4. The selected parameter set was number 63 (P1 = 4.5 and P2 = 9), where precision in terms of the range of obtained CIs was 0.49–1.36 ms and 0.29–1.60 ms for the six phantoms used for precision evaluation for the cardiac and liver sequences, respectively. The ADAPTS and MLE results from the phantom validation of accuracy for both the cardiac and liver sequences are shown in Figures 5 and 6. For the cardiac sequence, bias and variability (expressed as limits of agreement) for ADAPTS was 0.26 ± 4.23 ms, while MLE resulted in a bias and variability of 0.35 ± 4.63 ms. The liver sequence resulted in ADAPTS having bias and variability of −0.23 ± 1.69 ms, while bias and variability for the MLE was −0.22 ± 1.55 ms. The number of pixels within the drawn ROIs ranged from 194 to 200 pixels.

### Patient Study

All images and reconstructed T2* maps were of adequate quality for determination of T2* in both heart and liver. In one patient, however, the cardiac image quality was visually suboptimal due to breathing artifacts, albeit adequate for analysis, and was, therefore, included in further analysis. In this patient we also found the largest intraobserver difference of 3.96 ms (11%). The range of cardiac and liver T2* was 9.6–51.2 ms and 0.6–25.0 ms, respectively, using the ADAPTS method. The range of obtained uncertainty estimates for the ADAPTS method, expressed as coefficient of variation (the estimated standard deviation divided by the ROI T2* value), was 0.05–0.46 for cardiac and 0.01–0.27 for liver measurements. The number of pixels within the drawn ROI:s ranged from 53–561 pixels for liver images and 44–358 pixels for heart images.

#### T2* by the ADAPTS and MLE Methods

**Figure 7:**
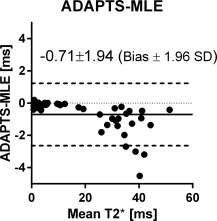
Bland‐Altman analysis of ADAPTS and MLE in patients measured by the experienced user. Good agreement was found.

The ADAPTS and the MLE methods showed good agreement determining T2*, resulting in a bias and variability with limits of agreement of −1.28 ± 2.19 ms for the cardiac sequence; −0.13 ± 0.38 ms for the liver sequence and −0.71 ± 1.94 ms for the cardiac and liver sequences combined (Fig. 7). T2* measured by the ADAPTS method ranged from 0.60–51.2 ms, while the MLE method ranged from 0.7−51.5 ms. A subtle trend toward higher T2* values was found for the MLE method compared with the ADAPTS method (Fig. 7) resulting in a statistically significant linear‐regression slope (*P* < 0.0001 for the null‐hypothesis). However, poor goodness of fit was found (r^2^ = 0.43).

#### Intraobserver Variability

**Figure 8:**
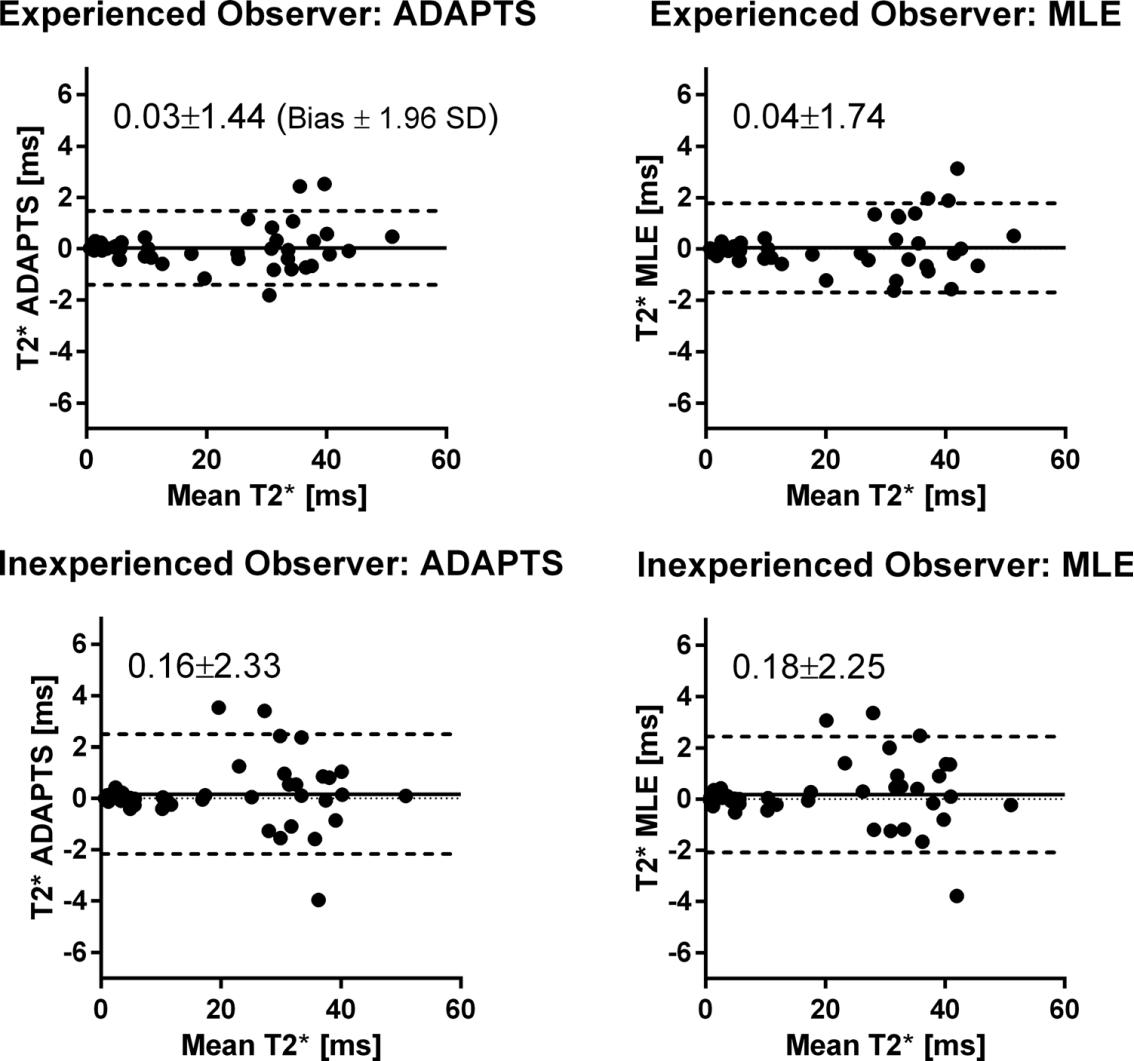
Bland‐Altman analyses of intraobserver variability for the experienced user for ADAPTS (top left) and MLE (top right). Corresponding analyses for the inexperienced user (bottom row). Good agreement was found between all measurements.

Low intraobserver variability was found for T2* determination by the experienced observer using both ADAPTS (0.12 ± 1.92 ms for the cardiac sequence, −0.06 ± 0.63 ms for the liver sequence and 0.03 ± 1.44 ms for both sequences combined; Figure 8, top left panel) and the MLE (0.20 ± 2.39 ms for the cardiac sequence, −0.12 ± 0.65 ms for the liver sequence and 0.04 ± 1.74 ms for both sequences combined; Figure 8, top right panel). Intraobserver variability was low also for the inexperienced observer using ADAPTS (0.01 ± 2.63 ms for the cardiac sequence, 0.31 ± 1.94 ms for the liver sequence and 0.16 ± 2.33 ms for both sequences combined; Figure 8, bottom left panel) and MLE (0.06 ± 2.66 ms for the cardiac sequence, 0.30 ± 1.85 ms for the liver sequence and 0.18 ± 2.25 ms for both sequences combined; Figure 8, bottom right panel) methods.

#### Interobserver Variability

**Figure 9:**
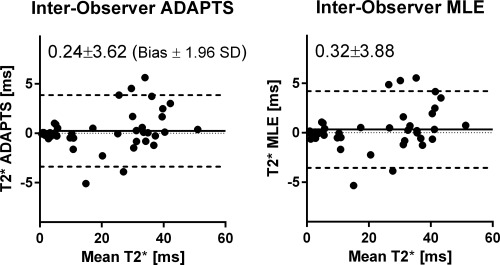
Bland‐Altman analysis of interobserver variability using the ADAPTS method (left panel) and MLE (right panel). Good agreement was found between the experienced and inexperienced observer.

Interobserver variability was low for both methods. Good agreement was found for both ADAPTS (limits of agreement of 1.0 ± 3.77 ms for the cardiac sequence, −0.52 ± 2.75 ms for the liver sequence and 0.24 ± 3.62 ms for both sequences combined; Figure 9, left panel) and MLE (limits of agreement of 1.17 ± 4.16 ms for the cardiac sequence, −0.53 ± 2.90 ms for the liver sequence and 0.32 ± 3.88 ms for both sequences combined; Figure 9, right panel).

## DISCUSSION

This study shows that the proposed automatic ADAPTS method provides accurate and precise determination of T2* in heart and liver for iron‐load quantification in a wide and clinically relevant range for use in an offline setting. The ADAPTS method provides uncertainty estimates of the calculated T2* value, which is of importance to assess the reported T2* validity, especially in follow‐up studies and for titrating treatment. The process of dividing pixels within the ROI into subregions to estimate uncertainty is not unique for ADAPTS and can be applied to most other T2* algorithms. However, validation both numerically and experimentally of such uncertainty measurements are crucial for clinical utility.

T2* determination by MRI is commonly used to estimate iron load in patients and has been shown to provide good interscanner and interobserver reproducibility 4, 5. The requirement for manual interaction in some of the current analysis methods adds a subjective factor which, although often clinically insignificant in myocardial T2* measurements 27, may present challenges in liver T2* determination 9, 19, 28. Iron overload is usually first found in the liver 29. Therefore, accurate early determination of liver iron load and treatment thereof may prevent accumulation of iron throughout the body and thus avert organ failure 30, 31, 32.

However, extremely low T2* values related to severe iron load of the liver may give few usable data points from the acquired images, due to current limitations in hardware to further reduce TE. This may lead to incorrect iron‐load assessment and possibly erroneous follow‐up of chelation therapy.

The automatic MLE method has been compared with other available methods for iron‐load determination, showing good agreement but also superiority for lower T2* values found in severe liver iron overload 19. By removing the need for manual curve‐fitting interaction, the MLE method decreases user dependency. Using the MLE method thereby allowed us to test ADAPTS's validity in patients with reduced user bias. We found that ADAPTS reports accurate T2* values with inter‐ and intraobserver variability comparable to the MLE method, and thereby can be reliably used, strengthened by phantom validation with the sGRE reference standard. In addition, ADAPTS was shown to have similar precision as a near‐optimal, noise correction method in numerical simulations. Although ADAPTS resulted in increased bias, the two‐parameter M2NCM used the true noise standard deviation generally unavailable to offline estimation methods. Compared with a single simulated receive‐coil, bias was increased for 6 and 32 coils in simulations. The observed increase in bias agrees with the expected increase in noise bias for the noncentral chi distribution present in the simulated RSS reconstruction 12. This indicates that a slight sensitivity to noise‐bias remains for ADAPTS. However, the observed bias were limited and converged when the number of coils increased from 6 to 32. An increase in precision for both T2* methods was observed when the number of coils increased. The gain in SNR associated with increasing the number of coils in RSS reconstruction may explain this 33.

The T2* uncertainty estimate gives the user possibility to evaluate the precision of measurement and helps determine whether changes in iron load levels have actually occurred between scans. This is especially important in high liver iron load which has a very steep T2* curve and where individual data points may have a large impact on reported T2* values.

Over‐ and underestimation of the reference T2* CI was observed in phantoms. This behavior was not seen in the numerical simulations where the proposed uncertainty estimate followed the reference CIs with low bias. The images acquired in the phantom experiments may include some degree of noise correlations between receive‐channels or adjacent pixels which could partly explain the discrepancy. Furthermore, the uncertainty estimate is sensitive to spatial variations in SNR and T2*. This, however, may aid the user in ROI adjustments by reporting elevated uncertainty in ROIs containing unwanted, heterogeneous T2* regions and noise levels.

Noteworthy, with decrease in iron load a statistically significant trend toward higher T2* values was found in patients for the MLE method compared with the ADAPTS. It remains to study why this happens, and more importantly the clinical significance of these differences. A retrospective study in a large population with biopsy samples available may help shed light on clinical significance and impact of cutoff values for severity of iron load.

Subtle differences were found between ADAPTS and MLE for intra‐ and interobserver variability. The sample size was, however, too small for deductions of increased performance in regards to user‐dependency. One major difference between MLE and ADAPTS is the pixelwise fit performed by the MLE. This may, in part, explain some of the observed discrepancies. Previous studies have shown decreased performance of other pixelwise methods 27, 34, 35, however, investigating the extent to which these findings are valid for the MLE method is beyond the scope of this study. Bias between observers and intraobserver variability for the inexperienced observer using the ADAPTS method were lower than previously published data 36. This implies that ADAPTS may be straight‐forward to start using in centers with low experience of iron load analyses, which may increase availability of iron load determination using MR imaging, in turn leading to enhanced patient care and further decrease of mortality 37.

### Limitations

A single‐slice approach was applied for patient imaging as this is clinical routine at one of the including centers. The algorithm is, however, not restricted to single‐slice acquisition and can be extended to multislice analysis where needed.

Simulations of spatially varying noise were not performed. Future simulation studies using advanced MRI pulse‐sequence simulations 38 may provide improvements in this regard.

## CONCLUSIONS

ADAPTS is a validated automatic algorithm for T2* determination providing accurate iron load measurements over a wide range of clinically relevant T2* values for the heart and liver. Uncertainty estimates of the reported T2* allows more reliable determination of changes in iron load at follow‐up. To allow practical utility of the method the software is freely available for research purposes. Phantom data will be made available upon request for algorithm benchmarking.

## Supporting information


**Figure S1**. Parameter optimization from simulations. Two near‐optimal values of P1 was simulated over the entire range of P2 values. Left column shows Confidence intervals (Top) and mean bias (bottom) for the simulated parameter values for the cardiac sequence TEs and the right column shows the corresponding plots for the liver sequence TEs. In all graphs, the solid lines and dashed lines represent simulations using one and six coils, respectively. The line marked with triangles indicates 32 simulated coils. The dotted vertical line shows the selected parameter set, corresponding to P1 = 4.5 and P2 = 9. A P1 value was selected by mainly considering stability, as shown in Figure 4.
**Figure S2**. Optimization of the uncertainty estimate in simulations with relative subregion sizes of 4–10%. Top row shows confidence intervals of the uncertainty estimates in simulations and the impact of varying simulated ROI‐size and subregion size percentages. Bottom row shows Mean bias of CI estimates with the left and right column showing the results from the cardiac and liver sequence TEs, respectively. Decreasing subregion percentages and increasing the ROI size improve precision of CI estimates. For the liver sequence TEs, bias is consistently decreased when the subregion size is reduced. The observed behavior is also seen in Supporting Figure S3.
**Figure S3**. Optimization of the uncertainty estimate in simulations with relative subregions sizes of 12–25%. Top row shows confidence intervals of the uncertainty estimates in simulations and the impact of varying simulated ROI‐size and subregion size percentages. Bottom row shows Mean bias of CI estimates with the left and right column showing the results from the cardiac and liver sequence TEs, respectively. Decreasing subregion percentages and increasing the ROI size improve accuracy and precision of CI estimates. The order of accuracy and precision among simulated subregion sizes is preserved over the evaluated ROI‐size interval. This may suggest that the relative percentages used to define the subregion size is robust to changes in ROI size. The observed behavior is also seen in Supporting Figure S2.
