# Supplementary material for: Validation of a new t2* algorithm and its uncertainty value for cardiac and liver iron load determination from MRI magnitude images
Source: Magn Reson Med. 2015 May 22;75(4):1717–29. doi: 10.1002/mrm.25767 (PMC4791092; doi:10.1002/mrm.25767)
Supplement: Supplementary file 1 — Figure S1. Parameter optimization from simulations. Two near‐optimal values of P1 was simulated over the entire range of P2 values. Left column shows Confidence intervals (Top) and mean bias (bottom) for the simulated parameter values for the cardiac sequence TEs and the right column shows the corresponding plots for the liver sequence TEs. In all graphs, the solid lines and dashed lines represent simulations using one and six coils, respectively. The line marked with triangles indicates 32 simulated coils. The dotted vertical line shows the selected parameter set, corresponding to P1 = 4.5 and P2 = 9. A P1 value was selected by mainly considering stability, as shown in Figure 4. Figure S2. Optimization of the uncertainty estimate in simulations with relative subregion sizes of 4–10%. Top row shows confidence intervals of the uncertainty estimates in simulations and the impact of varying simulated ROI‐size and subregion size percentages. Bottom row shows Mean bias of CI estimates with the left and right column showing the results from the cardiac and liver sequence TEs, respectively. Decreasing subregion percentages and increasing the ROI size improve precision of CI estimates. For the liver sequence TEs, bias is consistently decreased when the subregion size is reduced. The observed behavior is also seen in Supporting Figure S3. Figure S3. Optimization of the uncertainty estimate in simulations with relative subregions sizes of 12–25%. Top row shows confidence intervals of the uncertainty estimates in simulations and the impact of varying simulated ROI‐size and subregion size percentages. Bottom row shows Mean bias of CI estimates with the left and right column showing the results from the cardiac and liver sequence TEs, respectively. Decreasing subregion percentages and increasing the ROI size improve accuracy and precision of CI estimates. The order of accuracy and precision among simulated subregion sizes is preserved over the evaluated ROI‐size interval. This m [file MRM-75-1717-s001.docx]

Figure legends:

**Supporting Figure S1**: Parameter optimization from simulations. Two near-optimal values of P1 was simulated over the entire range of P2 values. Left column shows Confidence intervals (Top) and mean bias (bottom) for the simulated parameter values for the cardiac sequence echo times and the right column shows the corresponding plots for the liver sequence echo times. In all graphs, the solid lines and dashed lines represent simulations using 1 and 6 coils respectively. The line marked with triangles indicates 32 simulated coils. The dotted vertical line shows the selected parameter set, corresponding to P1 = 4.5 and P2 = 9. A P1 value was selected by mainly considering stability, as shown in Figure 4.





**Supporting Figure S2**: Optimization of the uncertainty estimate in simulations with relative sub-region sizes of 4-10%. Top row shows confidence intervals of the uncertainty estimates in simulations and the impact of varying simulated ROI-size and sub-region size percentages. Bottom row shows Mean bias of CI estimates with the left and right column showing the results from the cardiac and liver sequence echo times, respectively. Decreasing sub-region percentages and increasing the ROI size improve precision of CI estimates. For the liver sequence echo times, bias is consistently decreased when the sub-region size is reduced. The observed behavior is also seen in Supporting Figure S3.





**Supporting Figure S3**: Optimization of the uncertainty estimate in simulations with relative sub-regions sizes of 12-25%. Top row shows confidence intervals of the uncertainty estimates in simulations and the impact of varying simulated ROI-size and sub-region size percentages. Bottom row shows Mean bias of CI estimates with the left and right column showing the results from the cardiac and liver sequence echo times, respectively. Decreasing sub-region percentages and increasing the ROI size improve accuracy and precision of CI estimates. The order of accuracy and precision among simulated sub-region sizes is preserved over the evaluated ROI-size interval. This may suggest that the relative percentages used to define the sub-region size is robust to changes in ROI size. The observed behavior is also seen in Supporting Figure S2.
